# Ethnic Kawasaki Disease Risk Associated with Blood Mercury and Cadmium in U.S. Children

**DOI:** 10.3390/ijerph13010101

**Published:** 2016-01-05

**Authors:** Deniz Yeter, Michael A. Portman, Michael Aschner, Marcelo Farina, Wen-Ching Chan, Kai-Sheng Hsieh, Ho-Chang Kuo

**Affiliations:** 1 Kawasaki Disease Center, Kaohsiung Chang Gung Memorial Hospital, Niaosong, Kaohsiung 83301, Taiwan; wenching.chan@gmail.com (W.-C.C.); kshsieh@hotmail.com (K.-S.H.); 2 Division of Cardiology, Department of Pediatrics, Seattle Children’s Research Institute, University of Washington, Seattle, WA 98101, USA; michael.portman@seattlechildrens.org; 3 Department of Molecular Pharmacology, Albert Einstein College of Medicine, Yeshiva University, Bronx, NY 10461, USA; michael.aschner@einstein.yu.edu; 4 Departamento de Bioquímica, Centro de Ciências Biológicas, Universidade Federal de Santa Catarina, Florianópolis, Santa Catarina 88040, Brazil; marcelo.farina@ufsc.br; 5 Genomics and Proteomics Core Laboratory, Department of Medical Research, Kaohsiung Chang Gung Memorial Hospital, Niaosong, Kaohsiung 83301, Taiwan; 6 Department of Pediatrics, Kaohsiung Chang Gung Memorial Hospital, Niaosong, Kaohsiung 83301, Taiwan; 7 College of Medicine, Chang Gung University, Gueishan, Taoyuan 33302, Taiwan

**Keywords:** allergy, autoimmunity, infantile acrodynia, Kawasaki disease, mercury, methylmercury, pediatrics, pollution, seafood, toxicology

## Abstract

Kawasaki disease (KD) primarily affects children <5 years of age (75%–80%) and is currently the leading cause of acquired heart disease in developed nations. Even when residing in the West, East Asian children are 10 to 20 times more likely to develop KD. We hypothesized cultural variations influencing pediatric mercury (Hg) exposure from seafood consumption may mediate ethnic KD risk among children in the United States. Hospitalization rates of KD in US children aged 0–4 years (*n* = 10,880) and blood Hg levels in US children aged 1–5 years (*n* = 713) were determined using separate US federal datasets. Our cohort primarily presented with blood Hg levels <0.1 micrograms (µg) per kg bodyweight (96.5%) that are considered normal and subtoxic. Increased ethnic KD risk was significantly associated with both increasing levels and detection rates of blood Hg or cadmium (Cd) in a linear dose-responsive manner between ethnic African, Asian, Caucasian, and Hispanic children in the US (*p* ≤ 0.05). Increasing low-dose exposure to Hg or Cd may induce KD or contribute to its later development in susceptible children. However, our preliminary results require further replication in other ethnic populations, in addition to more in-depth examination of metal exposure and toxicokinetics.

## 1. Introduction

Kawasaki disease (KD) is an acute febrile illness predominately affecting children <5 years of age (75%–80%). KD patients develop a systemic autoimmune-like vasculitis leading to widespread inflammation of blood vessels and the small- to medium-sized arteries [1], which also demonstrates a predilection for the coronary arteries in particular. When left untreated, 20%–25% of KD patients develop coronary artery lesions (CAL) such as dilation, aneurysm, or fistula that can result in potentially fatal thrombosis or sudden cardiac failure [2]. Prompt detection of KD is required so that treatment is initiated with intravenous immunoglobulin (IVIG) before the 10th day after febrile onset to significantly reduce the risk of CAL formation in KD patients [3].

The cause of KD remains unknown and there is currently no test available to confirm its diagnosis. Therefore, KD continues to be diagnosed clinically through the exclusion of other similar illnesses. To satisfy a diagnosis of complete KD according to American Heart Association (AHA) guidelines [4], a patient must present with a high-grade fever that lasts 5 days or longer and is resistant to both antibiotics or antipyretics, in addition to four out of the following five principal diagnostic features: (1) a polymorphous rash; (2) changes in the extremities; (3) oral changes; (4) conjunctivitis; and (5) cervical lymphadenopathy. The principal symptoms of KD often develop over a week and do not always appear together. Incomplete cases are mostly diagnosed from the detection of CAL by echocardiogram [5].

Over the last few decades, KD has replaced rheumatic fever as the leading cause of acquired heart disease in the developed world. The highest rates of KD are observed in East Asia, particularly in Japan, Korea, and Taiwan where between 67.3 and 243.1 in 100,000 children <5 years develop the disease each year [6,7,8]. Even when residing in the West, East Asian children are 10 to 20 times more likely to develop KD than children of other ethnic backgrounds. KD rates are markedly lower in both Europe and North America (4.9–26.2 in 100,000) [9], while the highest rates of KD in the US are reported from the State of Hawaii (50.4 in 100,000) that has the largest predominate Asian population [10]. The incidence of KD has been increasing in East Asia and also appears to be increasing across the world.

This has led to speculation that the genetic variations between differing ethnic groups may account for the significant ethnic disparity in KD rates. Several susceptibility loci in KD have been identified through the meta-analysis of previous genome-wide association study (GWAS) investigations, such as *BLK*, *CASP3*, *CD40*, *FCGR2A*, *HLA-DOB*, and *ITPKC* [11,12,13,14,15,16,17,18]. However, these genetic susceptibility markers confer risk to KD development in a passive manner [19], and therefore sensitize individuals to the currently unknown environmental agents that induce KD in susceptible children [20]. As noted by Onouchi [19], the ethnic disparities in KD rates also “… *give the impression that KD is a type of endemic disease or a disease related to a lifestyle commonly found among the East Asian populations*.”

The clinical and epidemiological findings of KD that closely resemble an infectious disease have also given rise to suspicion that microorganisms could be causative agents of KD. Various bacterial and viral ailments such as measles or scarlet fever are often initially misdiagnosed in KD patients, which can lead to the unnecessary use of antibiotics or—most importantly—potential delay in required time-sensitive IVIG treatment. Subsequent efforts to isolate an infection since Kawasaki first established the illness internationally during the 1970s have been consistently met with conflicting results and no significant associations upon further replication. However, significantly higher rates of concurrent viral infections are observed in acute KD patients compared to healthy controls [21]. Researchers also note positive confirmation of pathogenic organisms in suspected KD should be interpreted with caution and not lead to immediate elimination of KD in the differential diagnosis [22,23,24], particularly detection via polymerase chain reaction (PCR).

Earlier authors initially suggested mercury (Hg) from pollution and seafood may be a cause of KD and account for the significantly higher rates of KD in Asian children [25,26]. These authors noted the striking clinical similarities between KD and infantile acrodynia, a childhood mercurial reaction that can mimic the complete clinical picture of KD and is therefore included in its differential diagnosis [5]. One reported case of acrodynia in a 13-year old female playing with elemental Hg fulfilled all six principal diagnostic findings for KD and was diagnosed after very high urine Hg levels were determined (580 µg/L; normal <10 µg in 24 h) [27]. Significantly elevated urine Hg levels from *n* = 6 acute KD patients compared to age-matched hospitalized controls (13.6 *vs.* 1.2 µg in 24 h; *p* = 0.018) had been previously reported by Orlowski and Mercer [28]. Two other investigations reported normal urine or hair Hg levels [29,30], although proper control groups were not used.

Previous examinations of Hg levels in the hair or urine of KD patients only involve a fairly small total of samples (*n* = 16). Recently, we reviewed how ITPKC susceptibility loci for KD would render individuals vulnerable to even low-level exposure to Hg [20], while Hg exposure can be difficult to detect in children through blood or urinalysis alone even when chronically exposed [31,32]. Therefore, we decided to investigate whether variations of Hg exposure among ethnic groups were associated with KD risk in US children <5 years of age by using blood Hg levels from a large sample of infants and young children aged 1–5 years in the general U.S. population.

## 2. Experimental Section

### 2.1. Study Cohorts

We retrieved the most recently published datasets from the U.S. Centers for Disease Control (CDC) that included demographic data for African-American, Asian, Caucasian, and Hispanic ethnicity. The Kids’ Inpatient Database (KID, [33]) was created in 1997 and is used by the U.S. CDC to estimate annual hospitalization rates of KD. The KID releases data every three years on approximately three million hospitalizations for children within the U.S. each year. The National Health and Nutrition Examination Survey (NHANES, [34]) was created in 1971 and is used by the U.S. CDC to monitor lead (Pb) levels in the blood of children. In 1999, the NHANES began to monitor Hg levels in the blood of children. Recently, self-reporting of Asian ethnicity was included for the first time in the latest NHANES cohort sample (2011–2012).

### 2.2. Kawasaki Disease Risk

KD risk was established through hospitalization rates of the illness per 100,000 using data from the KID that was published by the U.S. CDC. Weighted nationwide hospitalization rates of KD in U.S. children <5 years of age were retrieved for different reporting years from 1997 to 2007 and then averaged into a combined total. Nationwide KD hospitalization rates in U.S. children <5 years of age were stratified by ethnicity for each retrieved year and then respectively combined into an averaged total.

### 2.3. Blood Cadmium, Lead, Manganese, Mercury, Selenium

Detection rates and blood concentrations of Hg were retrieved from the 2011–2012 NHANES cohort of children aged 1 to 5 years in the general U.S. population that examined blood Hg, cadmium (Cd), lead (Pb), manganese (Mn), and selenium (Se). Blood levels below the lower detection limit were assigned by NHANES laboratories as a value of 0.11 µg/L for Cd or Hg, 1.61 µg/L for Mn, 0.18 µg/dL for Pb, and 96.24 µg/L for Se. Blood values of Cd, Hg, Mn, Pb, and Se were determined for our cohort population and then stratified by ethnicity.

### 2.4. Statistical Analysis

We determined statistical significance for each respective blood value for an ethnicity by comparing an individual ethnicity to the remaining cohort data using independent T-tests. ANOVA testing was conducted to determine overall significance between ethnicities. Blood values were then analyzed for linear dose-responsive associations with ethnic KD risk in a Pearson correlation test after the data had been stratified by ethnicity. To obtain statistical significance in any of our testing, we set a *p*-value of ≤ 0.05 for our results. Our blood values were weighted by ethnicity and used to set normalized values for the general U.S. population of children in this age bracket.

Weighted values were determined by using 2012 U.S. census data of self-reported ethnicity in children aged 0 to 18 years (African-American: 13.9%; Asian: 4.8%; Caucasian: 52.8%; Hispanic: 23.8%; Other: 4.7%), as data specifically for U.S. children aged 1 to 5 years could not be identified. Odds ratio (OR) values were determined for both of our cohorts and respectively examined ethnicities by comparing their blood values to our weighted values. Our subsequent analysis of ethnic KD risk did not include any values for children from the general U.S. population. Lastly, we constructed linear regression models of associations that were identified to be statistically significant using every individual reporting year for KD rates to create our figures and display a more accurate accounting of the ethnic variation.

## 3. Results

### 3.1. KID Cohort

Hospitalization rates of KD using the KID cohort were retrieved from the U.S. CDC for 1997, 2000, and 2006 [35,36]. Our cohort included a total of *n* = 14,193 diagnosed KD cases <18 years of age and *n* = 10,880 diagnosed KD cases <5 years of age (76.7%). As shown in Table 1, Caucasian children represented 46.7% of KD cases <5 years of age with a reported ethnicity (*n* = 3751) while 20.1% were Hispanic (*n* = 1614), 19.2% were African-American (*n* = 1540), 10.2% were Asian (*n* = 823), and 3.8% were Other (*n* = 307). Estimations of KD rates were not established for the indigenous American or Other ethnic categories as a result of low KD reporting and were therefore excluded from subsequent analyses. Ethnicity was missing for 26.1% of our KD cases <5 years of age and led to an underestimation of hospitalization rates among children for every ethnic group except Caucasians. As a result, weighted estimations for hospitalization rates of KD in the general U.S. population were subsequently underestimated. Sex was missing in 0.4% of KD cases <5 years of age (*n* = 41), while patient age was retrieved for each case.

**Table 1:** KID and NHANES cohort sample sizes and weighted ethnicity in U.S. children.

Ethnicity	KID (1997–2006) ^1^	NHANES (2011–2012) ^2^	U.S. Census (2012) ^3^
**African**	*n* = 1540 (19.2%)	*n* = 238 (33.4%)	*n* = 10,235,449 (13.9%)
**Asian**	*n* = 823 (10.2%)	*n* = 60 (8.4%)	*n* = 3,501,690 (4.8%)
**Caucasian**	*n* = 3751 (46.7%)	*n* = 119 (16.7%)	*n* = 38,915,681 (52.8%)
**Hispanic**	*n* = 1614 (20.1%)	*n* = 256 (35.9%)	*n* = 17,569,191 (23.8%)
**Other**	*n* = 307 (3.8%)	*n* = 40 (5.6%)	*n* = 3,486,168 (4.7%)
**TOTAL**	***n* = 8035 (73.9%)**	***n* = 713 (100%)**	***n* = 73,708,179**

Notes: ^**1**^ Age 0 to 4 years; ^**2**^ Age 1 to 5 years; ^**3**^ Age 0 to 18 years (children born in 1994–2012).

### 3.2. NHANES Cohort

Children aged 1 to 5 years accounted for 12.3% (*n* = 1203) of individuals enrolled into the 2011–2012 NHANES study (*n* = 9756). Of these children aged 1 to 5 years, *n* = 713 (59.3%) were further enrolled into the NHANES study examining blood Cd, Hg, Mn, Pb, and Se levels. Each child enrolled into our NHANES cohort was tested for all five of the studied elements. Sex was reported for every child included in our cohort, which contained *n* = 368 males (51.6%) and *n* = 345 females (48.4%). Ethnicity was also retrieved for every child included in our sample. No children under 1 year of age were included in the cohort data, which is likely a result of blood draw limitations in this vulnerable age group.

As shown in Table 1, 33.4% of cases were reported as non-Hispanic Black (*n* = 238), 22.0% as Mexican-American (*n* = 157), 16.7% as non-Hispanic white (*n* = 119), 13.9% as Other Hispanic (*n* = 99), 8.4% as non-Hispanic Asian (*n* = 60), and 5.6% as non-Hispanic multiracial (*n* = 40). For our study purposes, the Mexican-American and Other Hispanic categories were combined into a single group for Hispanic ethnicity. Lastly, non-Hispanic multiracial cases were designated as the Other ethnic category and then included in our weighted results, although we did not include them in any of our analysis for ethnic KD risk as a result of inadequate ethnic reporting from the KID cohort.

### 3.3. Kawasaki Disease Hospitalizations

As shown in Table 2, Asian children had the highest risk of KD hospitalization with 33.9 in 100,000 children <5 years annually developing the illness. Following Asians, African-American and Hispanic children presented with the next highest rates of KD with 18.0 and 13.5 in 100,000 children <5 years respectively developing the illness each year. By comparison, Caucasian children had the lowest rates with 10.8 in 100,000 children <5 years annually developing KD. The combined weighted total for annual KD hospitalization rates in the general U.S. population was 18.5 in 100,000 children <5 years. In total, Asian children had an 83% higher risk of developing KD, while Caucasian children presented with a 42% lower risk of developing KD compared to the general U.S. population <5 years of age.

**Table 2:** Kawasaki disease hospitalizations per 100,000 U.S. children aged 0 to 4 years.

Ethnicity	Averaged (OR)	1997 (OR)	2000 (OR)	2006 (OR)
**African**	18.0 (0.97)	16.9 (0.96)	19.7 (1.15)	17.5 (0.84)
**Asian**	33.9 (1.83)	32.5 (1.85)	39.0 (2.28)	30.3 (1.46)
**Caucasian**	10.8 (0.58)	9.1 (0.52)	11.4 (0.67)	12.0 (0.58)
**Hispanic**	13.5 (0.73)	11.1 (0.63)	13.6 (0.80)	15.7 (0.75)
**Other ^1^**	n/a	n/a	n/a	n/a
**Weighted**	**18.5**	**17.6 (0.95)**	**17.1 (0.92)**	**20.8 (1.12)**

Note: ^**1**^ Excluded from analyses as a result of low KD reporting.

### 3.4. Mean Blood Determinations

As shown in Table 3, children of Asian descent had the highest average levels of blood Cd (0.17 µg/L; *p* < 0.001), Hg (0.92 µg/L; *p* = 0.001), and Mn (12.69 µg/L; *p* < 0.001), while presenting with the lowest average levels of blood Pb (0.92 µg/dL; *p* < 0.001). African-American children presented with the highest average blood levels of Pb (1.63 µg/dL; *p* < 0.001), while presenting with the lowest average blood Mn levels (9.23 µg/L; *p* < 0.001). Caucasian children had the lowest average levels of blood Cd (0.12 µg/L; *p* < 0.001) and Hg (0.26 µg/L; *p* < 0.001). Further ANOVA testing demonstrated overall statistical significance between ethnicities for average blood levels of Cd, Hg, Mn, and Pb (*p* < 0.001). However, no significant findings were observed for blood Se.

**Table 3:** Average, minimum, and maximum values for blood cadmium, mercury, manganese, lead, and selenium in U.S. children aged 1 to 5 years.

Ethnicity	Cd µg/L ^2^ (OR)	Hg µg/L ^2^ (OR)	Mn µg/L ^3^ (OR)	Pb µg/dL ^4^ (OR)	Se µg/L ^5^ (OR)
	[min–max]	[min–max]	[min–max]	[min–max]	[min–max]
**African**	0.13 (1.00)	0.50 (1.39)	9.23 (0.84) **	1.63 (1.15) **	165.25 (0.99)
	[0.11–0.43]	[0.11–18.89]	[4.83–17.49]	[0.29–18.37]	[110.24–217.25]
**Asian**	0.17 (1.31) **	0.92 (2.56) **	12.69 (1.15) **	0.92 (0.65) **	165.00 (0.98)
	[0.11–0.39]	[0.11–5.18]	[5.94–25.42]	[0.29–3.26]	[102.34–204.90]
**Caucasian**	0.12 (0.92) **	0.26 (0.72) **	10.86 (0.98)	1.59 (1.12)	169.22 (1.01)
	[0.11–0.28]	[0.11–1.05]	[6.28–24.49]	[0.18–27.88]	[126.37–241.38]
**Hispanic**	0.13 (1.00)	0.38 (1.06) *	12.04 (1.09) **	1.07 (0.75) **	165.82 (0.99)
	[0.11–0.27]	[0.11–1.98]	[3.97–25.54]	[0.26–5.24]	[96.24–221.36]
**Other ^1^**	0.13 (1.00)	0.44 (1.22)	11.58 (1.05)	1.11 (0.78)	166.73 (1.00)
	[0.11–0.22]	[0.11–4.57]	[4.84–21.94]	[0.44–3.14]	[126.95–202.08]
**TOTAL ^6^**	0.13 (1.00) **	0.45 (1.25) **	10.93 (0.99) **	1.33 (0.94) **	**166.16 (0.99)**
	**[0.11–0.43]**	**[0.11–18.89]**	**[3.97–25.54]**	**[0.18–27.88]**	**[96.24–241.38]**
**Weighted**	**0.13**	**0.36**	**11.04**	**1.42**	**167.54**

Notes: ^**1**^ Excluded from analyses as a result of low KD reporting; ^**2**^ Lower limit: 0.12 µg/L; ^**3**^ Lower limit: 1.62 µg/L; ^**4**^ Lower limit: 0.19 µg/dL; ^**5**^ Lower limit: 96.25 µg/L; ^**6**^ ANOVA testing for statistical significance between ethnicities Statistical significance: * *p*-value ≤ 0.05; ** *p*-value ≤ 0.01.

Mean blood values in our total cohort sample were 0.13 µg/L for Cd, 0.45 µg/L for Hg, 10.93 µg/L for Mn, 1.33 µg/dL for Pb, and 166.16 µg/L for Se. After weighing our results by ethnicity, these values were 0.13 µg/L for Cd, 0.36 µg/L for Hg, 11.04 µg/L for Mn, 1.42 µg/dL for Pb, and 167.54 µg/L for Se in the blood of children aged 1 to 5 years from the general U.S. population. Asian children had 31% higher Cd and 156% higher Hg in the blood compared to our weighted values from the general U.S. population. African-American children presented with 15% higher Pb and 16% lower Mn in the blood compared to our weighted values. Lastly, as shown in Supplemental Tables S1 and S2, blood Se levels significantly increased with older age while blood Mn levels decreased. Furthermore, blood Mn levels were significantly higher in females than males. No other significant differences were observed with age or sex in our cohort.

### 3.5. Detectable Blood Determinations

As shown in Table 4, Asian children presented with the highest blood detection rates of Cd (46.7%; *p* = 0.002) and Hg (96.7%; *p* < 0.001), while Caucasian children presented with the lowest blood detection rates of Cd (10.9%; *p* < 0.001) and Hg (73.1%; *p* = 0.037). Blood Mn, Pb, and Se were detected in every child, except in Caucasian children for whom Pb was only detected in 98.3% of blood samples. ANOVA testing demonstrated statistical significance between ethnic groups for detectable blood Cd (*p* < 0.001), Hg (*p* <0.001), and Pb (*p* = 0.040).

**Table 4:** Blood values at or above detectable limits in US children aged 1 to 5 years.

Ethnicity	Cd % (OR) ^2^	Hg % (OR) ^2^	Mn % ^3^	Pb % (OR) ^4^	Se % ^5^
**African**	20.2% (1.22)	86.1% (1.12) **	100%	100% (1.01)	100%
**Asian**	46.7% (2.81) **	96.7% (1.26) **	100%	100% (1.01)	100%
**Caucasian**	10.9% (0.66) **	73.1% (0.95) *	100%	98.3% (0.99)	100%
**Hispanic**	19.9% (1.20)	77.0% (1.00)	100%	100% (1.01)	100%
**Other ^1^**	22.5% (1.36)	72.5% (0.94)	100%	100% (1.01)	100%
**TOTAL ^6^**	20.9% (1.26) **	80.8% (1.05) **	**100%**	99.7% (1.01) *	**100%**
**Weighted**	**16.6%**	**76.9%**	**100%**	**99.1%**	**100%**

Notes: ^**1**^ Excluded from analyses as a result of low KD reporting; ^**2**^ Lower limit: 0.12 µg/L; ^**3**^ Lower limit: 1.62 µg/L; ^**4**^ Lower limit: 0.19 µg/dL; ^**5**^ Lower limit: 96.25 µg/L; ^**6**^ ANOVA testing for statistical significance between ethnicities Statistical significance: * *p*-value ≤ 0.05; ** *p*-value ≤ 0.01.

Total blood detection rates were 20.9% for Cd, 80.8% for Hg, and 99.7% for Pb in our total cohort sample. After we weighted our results by ethnicity, blood detection rates were 16.6% for Cd, 76.9% for Hg, and 99.1% for Pb in the general U.S. population among children 1 to 5 years of age. Asian children had 181% higher rates of detectable Cd and 26% higher rates of detectable Hg in the blood compared to the general U.S. population using weighted values, while Caucasian children presented with 34% lower rates of detectable blood Cd. No significant differences were observed in blood detection rates between age or sex except for detectable Cd that was significantly lower in children aged 1 year, as shown in Supplementary Tables S3 and S4.

### 3.6. Linear Dose-Responsive Analysis

As shown in Table 5, we observed significant associations of increasing ethnic KD risk with increasing levels of blood Cd (*p* = 0.016) or Hg (*p* = 0.003) in a linear dose-responsive manner. Significant associations were identified of increasing ethnic KD risk with increasing detection rates of Cd (*p* = 0.016) or Hg (*p* = 0.033) in the blood. Further analysis demonstrated a significant association of increasing ethnic KD risk with the bottom or top percentiles of blood Hg (*p* = 0.042 and 0.014), in addition to the top percentiles of Cd in the blood (*p* = 0.002) that are viewable in Supplementary Table S5. Statistically significant associations were not observed for either blood Mn, Pb, or Se in any of our multiple testing.

**Figure 1:**
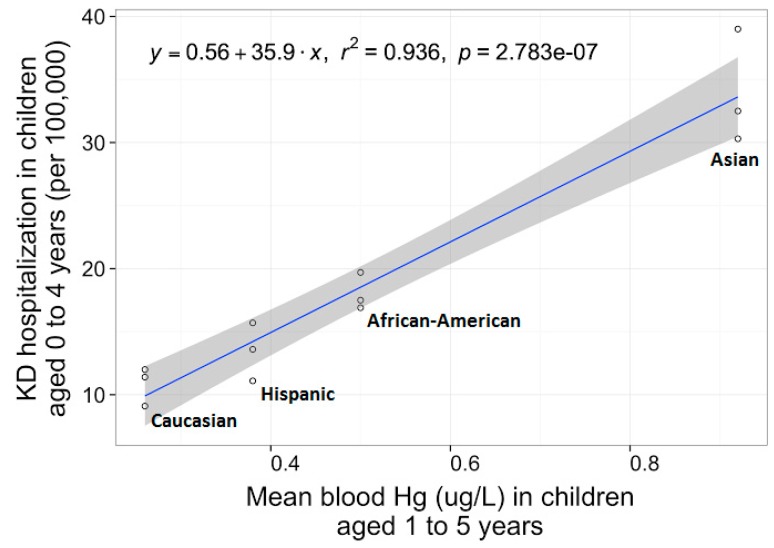
Blood mercury and ethnic Kawasaki disease risk. The logistic regression model for our initially identified association of increasing ethnic Kawasaki disease (KD) risk with higher average mercury (Hg) levels in the blood (*p* = 0.003) was also statistically significant (*p* < 0.001), and suggests Hg levels in the blood account for 93.6% of the ethnic variation in KD rates in a linear dose-responsive manner as demonstrated by the *r*^2^ value.

Blood Hg was the only variable that returned a value for each test and also remained statistically significant in all of our respective tests for ethnic KD risk. In addition, average levels of Hg in the blood also accounted for 99.5% of the ethnic variation in KD risk, which was the highest value for mean levels compared to average blood Cd levels (96.8%) as shown in Table 5. We also conducted a more reliable test using blood detection rates of Cd that accounted for 96.7% of the ethnic variation in KD rates after we observed that our mean blood Cd levels were close to or at the lowest detection limit (0.12–0.17 µg/L *vs.* 0.12 µg/L). Lastly, ethnic blood Hg levels displayed the most impressive linear dose-responsive association in our logistic regression model using retrieved means, as shown in Figure 1.

**Table 5:** Significant ethnic variation of KD risk in U.S. children accounted for by the *r*^2^ value from Pearson correlation tests.

Elements	Average µg/L	Detection Rate	Bottom Percentile	Top Percentile
**Cd**	96.8% *	96.7% *	n/a ^2,3^	99.6% **^,4^
**Hg**	99.5% **	93.6% *	91.7% *^,3^	97.2% *^,4^
**Mn**	22.5%	n/a ^1^	0.1% ^3^	21.0% ^4^
**Pb**	40.4%	28.3%	0.8% ^3^	43.0% ^5^
**Se**	42.4%	n/a ^1^	12.6% ^3^	55.8% ^4^

Notes: ^**1**^ 100% detection; ^**2**^ No difference in values; ^**3**^ 5th percentile; ^**4**^ 95th percentile; ^**5**^ 97.5th percentile; Statistical significance: * *p*-value ≤ 0.05; ** *p*-value ≤ 0.01.

## 4. Discussion

KD is an autoimmune-like vasculitis driven by markedly elevated inflammatory and immune cells. An infectious agent has not been consistently isolated in KD and the cause of KD remains unknown despite nearly half a century of extensive international investigation. The prevailing theory for causation involves a pathogen or other environmental agents that trigger an allergic or hypersensitivity type of response, which only results in clinical KD among susceptible children through mediating factors such as genetics and possibly other idiosyncrasies. Serum immunoglobulin E (IgE) and interleukin-4 (IL-4) are significantly elevated in acute KD patients [37], which supports a hypersensitivity theory. IgE and IL-4 levels are used as biomarkers for the severity of asthma and allergic diseases. In addition, children with KD are significantly more likely to develop asthma and common allergies both before and after the onset of KD, while children with an onset of allergic diseases during childhood are also significantly more likely to develop KD [38,39]. Pollution and passive exposure to environmental tobacco smoke are well-known for their associations with the development and exacerbation of asthma and allergic diseases [40,41,42,43]. Seasonal pollen exposure was recently observed to be associated with KD in Japan and attributed as a delayed hypersensitivity reaction [44].

Rodó *et al.* [45] previously reported seasonal shifts in large-scale wind currents originating from China and Central Asia significantly associated with downstream KD cases in Japan, Hawaii, and Southern California. In their most recent study, yeast pollution from the soil of cereal crops in the heavy agricultural and industrial region of Manchuria in China appeared to account for the significant association of KD cases in neighboring Japan [46], which suggests KD is caused by pre-formed toxins. Metal contamination with Cd and Hg in particular is one of the most pressing issues currently facing soil quality in China as a result of rapid industrialization [47]. Previously, the contamination of soil for rice crops with Cd from mining pollution resulted in an appearance of Itai-Itai disease in Japan from the 1940s to the 1960s [48]. Local seafood highly contaminated with methylmercury led to the initial appearance of Minamata disease in Japan during the 1950s and the 1960s [49], which also corresponds to the initial appearance of KD in Japan after the end of the 1940s during the 1950s and 1960s [50]. Lastly, toxic metals impact soil microbiota and yeast growth [51].

There is no physiological role of Cd, Hg, or Pb in the body, which are considered to have no safe doses for exposure and are of notable public health concern, particularly in young children who are most susceptible [52]. Hypersensitivity or idiosyncratic reactions to Hg such as infantile acrodynia often occur at levels considered to be normal, low, or subtoxic doses [31,32]. We observed that increased blood Hg was significantly associated with increasing ethnic KD risk (Figure 1), although our average blood Hg levels in all ethnic groups are considered to be normal or subtoxic (<0.1 µg Hg intake per kg bodyweight per day) [53]. Researchers now stress the use of Hg and Se molar ratios in blood or examined tissues to more precisely determine methylmercury toxicity from seafood consumption, as there appears to be a direct relation between the toxicity of methylmercury and Se content [54]. However, our average blood Se levels did not significantly differ between ethnicity in our current study, while decreasing Hg exposure appeared to be far more important than increasing Se intake as has been previously reported by other authors [55].

We used total Hg in the blood as it reflects recent Hg exposure, particularly from fish consumption. The half-life of methylmercury is much longer in the blood than other forms of Hg such as the inorganic forms [56], while methylmercury in the blood is converted into other forms of Hg after its absorption in the body. Asian children in the U.S. between 1 and 2 years of age have the highest average dietary intake of methylmercury by body weight per day compared to the rest of the U.S. population as a result of significantly higher rates of seafood consumption [57], which also parallels the peak age of incidence for KD in East Asia [6,7,58], in addition to the US [10,36]. Dietary seafood intake significantly increases Hg levels in the blood of young children [54,59], particularly large predatory fish such as tuna, swordfish, and shark that contain the highest methylmercury levels as a result of aquatic bioaccumulation. Our results suggest that the supplementation of large predatory fish with seafood that instead contains lower levels of Hg may be preferable, particularly in vulnerable populations such as young children and the developing fetus.

This current study is accompanied by certain limitations. Hospitalization rates for KD were underestimated as a result of underreporting in all ethnicities except for Caucasians. In addition, demographic data regarding self-reported ethnicity from the KID and NHANES cohorts is generalized and does not elaborate on specific Asian ethnicity, even though East Asians have the highest KD risk even compared to other Asian ethnicities. Our investigation involved separate cohorts for KD risk or blood values from two different time periods, and therefore did not examine Hg levels or other blood samples in KD patients. We also did not include a complete accounting of KD rates in the U.S. since 1997 and instead included the available reporting years for 1997, 2000, and 2006 that were then subsequently averaged together. However, our collected KD rates should accurately reflect the current incidence once combined, as KD rates have remained relatively stable in the U.S. from 1997 to 2007 [36]. In addition, average blood Hg levels in children aged 1 to 5 years have not changed significantly since 1999 [60], which is when the NHANES first began testing for this metal.

Our current study was modeled after our previous examination of ethnic KD risk with the consumption of soy and isoflavones [61], which could possibly be mediated by FCGR2A susceptibility in KD [62]. However, we did not examine the impact of dietary soy or seafood consumption upon ethnic KD risk among children in either of our cohorts. Prenatal and postnatal sources of Hg exposure were also not examined in our cohorts. Blood Hg was not examined in women who are pregnant or of childbearing age, although it is recognized that exposure to methylmercury from seafood consumption and other forms of Hg cross into the placenta and result in prenatal exposure [63]. Blood values from the NHANES are not determined in children during their first year of life, which is likely a result for blood draw limitations in this age group. In addition, we were not able to examine the role of genetic susceptibility markers in either of our cohorts. Average blood Cd was considered too close to the lower detection limit in our cohort (0.12–0.17 µg/L *vs.* 0.12 µg/L), which then led us to conduct a more reliable examination by using detection rates of Cd in the blood. Lastly, our findings require additional replication with larger samples of KD patients and controls, including in other ethnic and regional populations.

As this study involves a population-based comparison of two separate datasets for KD risk or blood levels respectively in the general U.S. population, we can only currently afford speculation with regards to potential mechanisms or dietary factors that may link exposure to Cd and Hg with ethnic KD risk. Several animal studies have demonstrated that even low-level exposure to mercurials induces autoimmune vasculitis in susceptible rodent strains [64,65,66], which is characterized by T cell-dependent polyclonal activation of B lymphocytes, marked increases of serum immunoglobulins such as IgE and proinflammatory cytokines such as TNFα, detectable antinucleolar autoantibodies (ANoA), and widespread vascular immune complex deposition that may also be specific to various target organs such as in Hg-induced glomerulonephritis [67,68,69,70]. Many of these processes are also observed in KD, although certain investigations are limited in KD patients and still require further study. In addition, certain reported mechanisms of Hg-induced autoimmunity in animal models still require verification in humans. This has led several researchers to conduct examinations of Hg-exposed populations from seafood consumption or mining in affected regions, including among workers and local communities in Amazonian Brazil [71,72,73,74]. Some of these authors have further reported interactions between Hg exposure and infectious agents, as we also previously reviewed regarding animal models and synergistic toxicity between metals [20].

We suggest a more in-depth study of Cd and Hg toxicokinetics conducted in a large pediatric population with a high burden of KD risk that is multiethnic and primarily Asian. Such a prospective study should include parameters of cumulative exposure in addition to paired blood, urine, and hair levels, as blood Hg levels primarily represent recent exposure while urine Hg levels largely reflect current excretion and hair Hg levels mostly represent cumulative long-term exposure. The highly erratic nature of Hg levels in infantile acrodynia often results in dramatic day-to-day variation in blood and urine samples, which has led many authors to emphasize the need of 24-h collections for urine determinations of Hg and an observation of their trends over the span of several weeks or months [75]. Lastly, sources of Hg exposure in acrodynia patients are often occult or remain unidentified, which results from the myriad of exposure sources to this toxic metal in pediatric populations. Although pollution and the contamination of seafood and cereal crops may account for the primary exposure source in the general or patient population—lack of controlling for other Hg exposure sources or paired biomarkers in the blood, urine, and hair may result in the inability to significantly identity true associations that then become masked in the data.

## 5. Conclusions

In conclusion, we observed that exposure to Cd and Hg appears to be associated with an increasing ethnic KD risk, which may result from soil contamination of cereal rice crops with Cd or the contamination of seafood with methylmercury. KD is believed to be caused by widespread environmental agents that only induce the illness in susceptible children. In our current study, we observed that Cd or Hg at a normal and subtoxic level in the blood may induce KD young children, which may also be mediated by genetic susceptibilities and other factors at comparatively low doses of exposure. Examinations of blood and urine Hg levels in KD patients and sources of Hg exposure are required in large samples using febrile and healthy age-matched controls, as Hg levels vary greatly from day-to-day, between individuals, and even in patients with acrodynia. Exposed individuals and patients may present with normal Hg levels while the source of exposure remains unknown despite exhaustive investigation. Lastly, our findings still require further replication in other regional populations and ethnic groups.

## Supplemental Materials

Table S1. Average, minimum, and maximum values for blood cadmium, mercury, manganese, lead, and selenium in U.S. children aged 1 to 5 years by biological sex. Table S2. Average, minimum, and maximum values for blood cadmium, mercury, manganese, lead, and selenium in U.S. children aged 1 to 5 years by age. Table S3. Detection rates for blood cadmium, mercury, manganese, lead, and selenium in U.S. children aged 1 to 5 years by biological sex. Table S4. Detection rates for blood cadmium, mercury, manganese, lead, and selenium in U.S. children aged 1 to 5 years by age. Table S5. Bottom and top percentiles for blood cadmium, mercury, manganese, lead, and selenium in U.S. children aged 1 to 5 years.
