# Supplementary material for: Ethnic Kawasaki Disease Risk Associated with Blood Mercury and Cadmium in U.S. Children
Source: Int J Environ Res Public Health. 2016 Jan 5;13(1):101. doi: 10.3390/ijerph13010101 (PMC4730492; doi:10.3390/ijerph13010101)
Supplement: Supplementary file 1 [file ijerph-13-00101-s001.pdf]

Supplementary Materials: Ethnic Kawasaki Disease Risk Associated with Blood Mercury and Cadmium in U.S. Children

Deniz Yeter, Michael A. Portman, Michael Aschner, Marcelo Farina, Wen-Ching Chan, Kai-Sheng Hsieh and Ho-Chang Kuo

Table S1. Average, minimum, and maximum values for blood cadmium, mercury, manganese, lead, and selenium in US children aged 1 to 5 years by biological sex.

Sex	Cd μg/L [1] [min–max]	Hg μg/L [1] [min–max]	Mn μg/L [2] [min–max]	Pb μg/dL [3] [min–max]	Se μg/L [4] [min–max]
**Female**	0.13 [0.11–0.43]	0.44 [0.11–4.00]	**11.25** * [4.83–25.54]	1.22 [0.18–5.98]	165.63 [102.34–217.25]
**Male**	0.13 [0.11–0.34]	0.46 [0.11–18.89]	**10.63** * [3.97–21.94]	1.44 [0.18–27.88]	166.66 [96.25–241.38]
**TOTAL [5] (*n* = 713)**	**0.13 [0.11–0.43]**	**0.45 [0.11–18.89]**	**10.93** * **[3.97–25.54]**	**1.33 [0.18–27.88]**	**166.16 [96.24–241.38]**

Notes: [1] Lower limit: 0.12 μg/L; [2] Lower limit: 1.62 μg/L; [3] Lower limit: 0.19 μg/dL; [4] Lower limit: 96.25 μg/L; [5] ANOVA testing for statistical significance by biological sex, age, or ethnicity; Statistical significance: * p-value ≤ 0.050.

Table S2. Average, minimum, and maximum values for blood cadmium, mercury, manganese, lead, and selenium in US children aged 1 to 5 years by age.

Age	Cd μg/L [1] [min–max]	Hg μg/L [1] [min–max]	Mn μg/L [2] [min–max]	Pb μg/dL [3] [min–max]	Se μg/L [4] [min–max]
**1 Year**	**0.12** ** [0.11–0.28]	0.40 [0.11–4.57]	**11.90** ** [6.08–24.49]	1.42 [0.18–6.43]	**159.98** ** [110.24–209.96]
**2 Years**	0.13 [0.11–0.43]	0.50 [0.11–18.89]	10.73 [4.83–25.42]	1.58 [0.27–27.88]	**162.24** ** [96.24–215.84]
**3 Years**	0.13 [0.11–0.32]	0.44 [0.11–4.00]	11.24 [3.97–21.94]	1.31 [0.28–15.58]	166.33 [114.58–209.31]
**4 Years**	0.13 [0.11–0.39]	0.49 [0.11–5.18]	**10.44** * [4.84–25.54]	1.20 [0.26–5.02]	**170.98** ** [129.71–241.38]
**5 Years**	0.13 [0.11–0.34]	0.40 [0.11–2.44]	10.58 [4.89–20.35]	1.09 [0.18–5.88]	**171.69** ** [127.34–221.36]
**TOTAL [5]**	**0.13 [0.11–0.43]**	**0.45 [0.11–18.89]**	**10.93** ** **[3.97–25.54]**	**1.33 [0.18–27.88]**	**166.16** ** **[96.24–241.38]**

Notes: [1] Lower limit: 0.12 μg/L; [2] Lower limit: 1.62 μg/L; [3] Lower limit: 0.19 μg/dL; [4] Lower limit: 96.25 μg/L; [5] ANOVA testing for statistical significance by biological sex, age, or ethnicity; Statistical significance: * p-value ≤ 0.050; ** p-value ≤ 0.010.

Table S3. Detection rates for blood cadmium, mercury, manganese, lead, and selenium in US children aged 1 to 5 years by biological sex.

Sex	Detectable Blood Cd [1]	Detectable Blood Hg [1]	Detectable Blood Mn [2]	Detectable Blood Pb [3]	Detectable Blood Se [4]
**Female**	19.4%	80.9%	100%	99.7%	100%
**Male**	22.3%	80.7%	100%	99.7%	100%
**TOTAL [5]**	**20.9%**	**80.8%**	**100%**	**99.7%**	**100%**

Notes: [1] Lower limit: 0.12 μg/L; [2] Lower limit: 1.62 μg/L; [3] Lower limit: 0.19 μg/dL; [4] Lower limit: 96.25 μg/L; [5] ANOVA testing for statistical significance by biological sex, age, or ethnicity.

Table S4. Detection rates for blood cadmium, mercury, manganese, lead, and selenium in US children aged 1 to 5 years by age.

Age	Detectable Blood Cd [1]	Detectable Blood Hg [1]	Detectable Blood Mn [2]	Detectable Blood Pb [3]	Detectable Blood Se [4]
**1 Year**	**13.0% ****	78.3%	100%	99.1%	100%
**2 Years**	21.2%	79.9%	100%	100%	100%
**3 Years**	22.7%	80.9%	100%	100%	100%
**4 Years**	25.4%	81.5%	100%	100%	100%
**5 Years**	20.5%	83.5%	100%	99.2%	100%
**TOTAL [5]**	**20.9%**	**80.8%**	**100%**	**99.7%**	**100%**

Notes: [1] Lower limit: 0.12 μg/L; [2] Lower limit: 1.62 μg/L; [3] Lower limit: 0.19 μg/dL; [4] Lower limit: 96.25 μg/L; [5] ANOVA testing for statistical significance by biological sex, age, or ethnicity; Statistical significance: * p-value ≤ 0.050; ** p-value ≤ 0.010.

Table S5. Bottom and top percentiles for blood cadmium, mercury, manganese, lead, and selenium in US children aged 1 to 5 years.

Ethnicity	Cd μg/L [2] Bottom [6] (OR) Top [7] (OR)	Hg μg/L [2] Bottom [6] (OR) Top [7] (OR)	Mn μg/L [3] Bottom [6] (OR) Top [7] (OR)	Pb μg/dL [4] Bottom [6] (OR) Top [8] (OR)	Se μg/L [5] Bottom [6] (OR) Top [7] (OR)
**African**	0.11 (1.00)	0.11 (1.00)	5.42 (0.81)	0.58 (1.38)	137.77 (1.01)
	0.23 (1.10)	1.23 (1.21)	13.83 (0.79)	5.40 (1.04)	198.59 (0.99)
**Asian**	0.11 (1.00)	0.18 (1.64)	6.79 (1.02)	0.38 (0.90)	134.55 (0.99)
	0.32 (1.53)	3.72 (3.65)	20.21 (1.15)	2.75 (0.53)	196.74 (0.98)
**Caucasian**	0.11 (1.00)	0.11 (1.00)	6.98 (1.04)	0.38 (0.90)	137.00 (1.01)
	0.20 (0.95)	0.54 (0.53)	17.65 (1.00)	6.43 (1.24)	202.92 (1.01)
**Hispanic**	0.11 (1.00)	0.11 (1.00)	6.92 (1.04)	0.39 (0.93)	133.96 (0.99)
	0.21 (1.00)	1.19 (1.17)	18.36 (1.04)	3.21 (0.62)	198.27 (0.99)
**Other [1]**	0.11 (1.00)	0.11 (1.00)	5.63 (0.84)	0.49 (1.17)	127.83 (0.94)
	0.21 (1.00)	2.10 (2.06)	21.26 (1.21)	3.12 (0.60)	195.31 (0.97)
**TOTAL**	**0.11 (1.00)**	**0.11 (1.00)**	**6.17 (0.92)**	**0.42 (1.00)**	**136.00 (1.00)**
	**0.23 (1.10)**	**1.27 (1.25)**	**17.20 (0.98)**	**4.08 (0.79)**	**199.85 (1.00)**
**Weighted**	**0.11**	**0.11**	**6.68**	**0.42**	**135.83**
	**0.21**	**1.02**	**17.58**	**5.18**	**200.56**

Notes: [1] Excluded from analyses as a result of low KD reporting; [2] Lower limit: 0.12 μg/L; [3] Lower limit: 1.62 μg/L; [4] Lower limit: 0.19 μg/dL; [5] Lower limit: 96.25 μg/L; [6] 5th percentile; [7] 95th percentile; [8] 97.5th percentile.

© 2016 by the authors; licensee MDPI, Basel, Switzerland. This article is an open access article distributed under the terms and conditions of the Creative Commons by Attribution (CC-BY) license (http://creativecommons.org/licenses/by/4.0/).
